# Defining terminology and outcome measures for evaluating overdose response technology: An international Delphi study

**DOI:** 10.1111/dar.14055

**Published:** 2025-04-25

**Authors:** William Rioux, Dylan Viste, Navid Sedaghat, Nathan Rider, Joseph Tay Wee Teck, Melissa Perri, David G. Schwartz, Kim Ritchie, Giuseppe Carrà, Stephanie Carreiro, Oona Kreig, Gabriela Marcu, Nicholas Bartlett, Joseph Arthur, Joanne Cogdell, Mike Brown, Tyler Marshall, S. Monty Ghosh

**Affiliations:** ^1^ Department of Medicine Faculty of Medicine & Dentistry, University of Alberta Edmonton Canada; ^2^ Department of Medicine Cumming School of Medicine, University of Calgary Calgary Canada; ^3^ Population and Behavioural Science School of Medicine, University of St Andrews St Andrews UK; ^4^ Dalla Lana School of Public Health, University of Toronto Toronto Canada; ^5^ Information Systems Division Graduate School of Business, Bar‐Ilan University Ramat‐Gan Israel; ^6^ Department of Social Work McMaster University Hamilton Canada; ^7^ Department of Medicine and Surgery University of Milano‐Bicocca Monza Italy; ^8^ Division of Psychiatry University College London London UK; ^9^ Department of Emergency Medicine Division of Medical Toxicology Worcester USA; ^10^ Brave Technology Co‐op Vancouver Canada; ^11^ School of Information, University of Michigan Ann Arbor USA; ^12^ Department of Asian and Middle Eastern Cultures Barnard College; ^13^ Department of Palliative, Rehabilitation and Integrative Medicine The University of Texas MD Anderson Cancer Houston USA; ^14^ Naxos Neighbors South Bend USA; ^15^ Never Use Alone McMinnville USA; ^16^ Department of Internal Medicine Faculty of Medicine & Dentistry, University of Alberta Edmonton Canada

**Keywords:** Ehealth, mobile overdose response services, overdose detection technologies, virtual harm reduction, virtual supervised consumption

## Abstract

**Introduction:**

Various novel harm reduction services leverage technology to reduce the rising number of drug poisoning deaths, particularly among those who use drugs alone. There is significant variability in terminology and outcome measures in reporting these interventions, complicating efforts to build a comprehensive knowledge base. Thus, we conducted a Delphi study to establish consensus and heterogeneity in these metrics.

**Methods:**

Panellists from three stakeholder groups (people who use drugs, virtual harm reduction service operators and academics) participated in a multi‐round Delphi study. The first round included open‐ended questions to propose items in three categories: terminology, demographic information and outcomes. Subsequent rounds included options from a previously conducted scoping review for consideration. Likert ratings were used to achieve consensus, with a 70% threshold. Final rounds involved ranking terminology that reached a consensus.

**Results:**

Of 23 initial participants, 14 completed the fourth survey round. “Overdose response technology” was identified as the most appropriate term for these harm reduction technologies. This definition includes drug contamination alerts, overdose response hotlines and applications, wearable overdose detection technology and overdose detection tools. Fourteen demographic outcomes reached a consensus for data collection, including name or handle, neighbourhood, age, gender, past overdose experience, substance used, amount and route of use. Six service use outcomes were recommended: response type, service outcomes, morbidity and mortality, overdose events, responder arrival time and post‐rescue care.

**Discussion and Conclusions:**

The study results are recommended to standardise terminology and guide future research and knowledge dissemination in the field, ensuring clear communication with a shared language.

## 1 INTRODUCTION

The world currently faces an unprecedented opioid crisis, which has claimed the lives of over a hundred thousand individuals globally in 2019, with certain regions—particularly North America—experiencing disproportionately high rates of opioid‐related harms [1]. While this is a multifaceted issue, evidence suggests that using drugs alone in combination with the toxic drug supply and lack of access to treatment and harm reduction supports are major contributors to the toll of this epidemic [2, 3, 4, 5]. To address this, a variety of novel digital and mobile health interventions have emerged to help reduce the harms associated with this crisis and act as adjuncts to current harm reduction services [6, 7, 8, 9, 10, 11]. It is thought that these various technologies, which include mobile applications, telehealth and telephone hotlines, may fill gaps resulting from limitations of in‐person supervised consumption sites [12, 13]. The majority of these novel harm reduction technologies provide access to more timely emergency responses in the event of overdoses or drug poisonings or disseminating public health information, such as drug contamination alerts. Indeed, these various services are thought to have the potential to expand harm reduction access to populations who have historically had little to no access, such as rural, remote and Indigenous communities [5, 14, 15, 16]. Recent evidence from one service has demonstrated that 68.2% of service users lacked access to any other harm‐reduction services in their area [17]. These services may also be lifesaving for those in urban settings who face stigma and are unable or unwilling to access in‐person services [9, 14].

A recent scoping review around virtual addiction care found three studies using digital health technologies to promote treatment access and three studies that used such technologies for overdose monitoring or prevention [6]. In a 2023 systematic review, Loverock et al. identified 40 studies involving digital health interventions delivering harm reduction services [8]. Across these individual studies, there was a lack of consistency in reporting terminology. Among the diverse terminology for the interventions were terms, such as “virtual overdose monitoring services”, “electronic harm reduction interventions”, “remote overdose monitoring services” and “overdose detection technologies”, among others [7, 8, 10, 11]. Having clarity in the definition, terminology, scope and necessary components of a complex intervention is a necessary first step to its successful implementation and evaluation [18]. This is particularly important in the case of technologies used to reduce the harms associated with the overdose crisis, as it is a relatively new field of research with a steep learning curve for researchers, policymakers, harm reduction service operators and people who use drugs (PWUD). Establishing a consensus on terminology and definitions may help these groups more easily identify and conceptualise different services. Furthermore, common terminology facilitates access, acceptability, effectiveness and service adoption discussions. Sharing common terminology facilitates knowledge‐sharing across different services and jurisdictions regarding funding models, evidence‐based quality standards, service delivery, ethical challenges and client privacy [18].

In addition to terminology, many overdose response technologies differ with regard to evaluation methods and outcome measures collected and reported [8, 11]. Collection of demographic data, consisting of descriptions of users of these services, and outcome data, including measures to determine the efficacy, safety and effectiveness of these services, are an essential part of harm reduction program evaluation. However, in the context of PWUD using harm reduction services, the collection of this data can be perceived as intrusive surveillance, stigmatising and a threat to anonymity, potentially acting as a barrier to their uptake of these interventions [19, 20]. The issue of demographic data collection and outcome measurement is, therefore, a particularly nuanced one, and has been hampered by significant heterogeneity in approach across the identified studies [6, 7, 8, 9, 10, 11, 12]. Consequently, a multi‐stakeholder discourse centred on PWUD perspectives on the standardisation of metrics for overdose response technology program evaluation is critical [20].

To address these issues, the aims of our study are twofold. First, the study aims to define and delineate the various types of digital health technologies for overdose response available to people who use drugs. Second, we aim to establish a core outcome set of standardised demographic and health outcomes that may be collected across all overdose response technologies. These outcomes could be used for program evaluation and quality improvement, but also research to inform relevant interest groups.

## 2 METHODS

**FIGURE 1:**
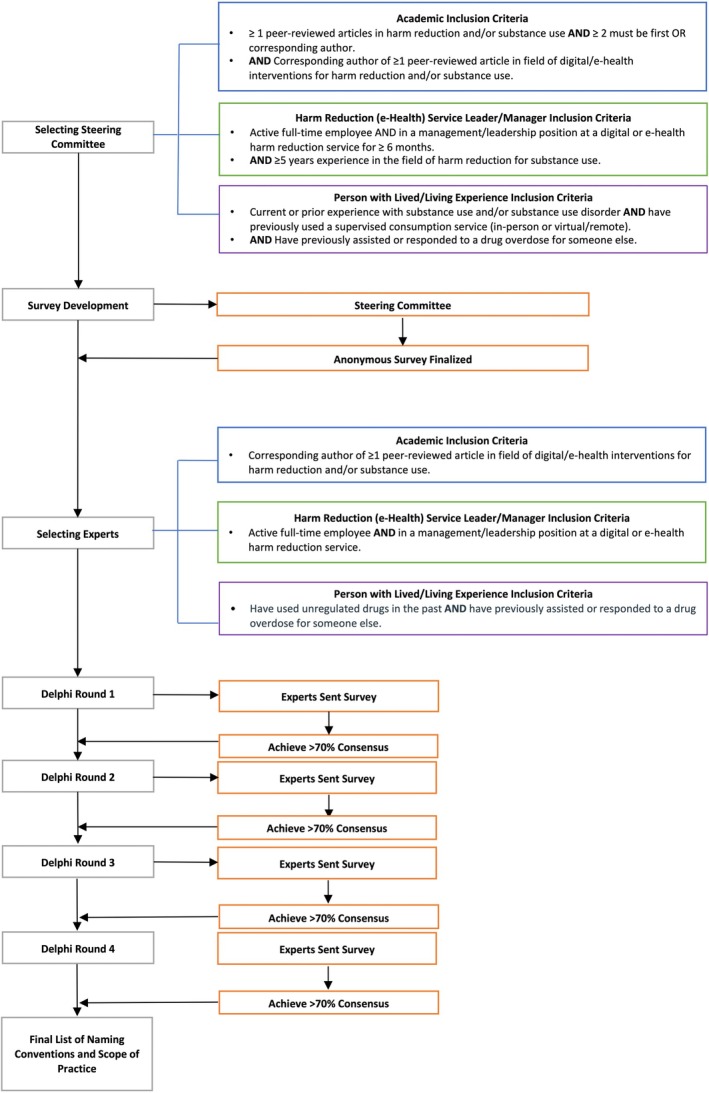
Delphi flow chart.

The Delphi methodology was registered on the Open Science Framework and the Core Outcome Measures in Effectiveness Trials (COMET) databases [21, 22]. The design and reporting of the study results were guided by Sinha et al. [23], Beiderbeck et al. [24] and the Guidance on Conducting and Reporting Delphi Studies (CREDES) guidelines [25]. Ethics approval was obtained from the University of Calgary (REB22‐1252). An overview of the entire Delphi process can be found in Figure 1.

### 2.1 Evidence review

A scoping review was conducted according to the PRISMA‐ScR and PRISMA for Searching guidelines from inception until 20 January 2023, the results of which have been previously published elsewhere [8]. The review identified 21 discrete technology‐based harm reduction measures which aim to reduce rates of fatal overdose across both peer‐reviewed and grey literature. Across the 21 identified technologies, a range of overlapping harm reduction interventions were described with diverse reporting of demographic and outcome data and terminology [8].

### 2.2 Participants

#### 2.2.1 Study participants

Study participants were recruited from 3 different demographic groups, including academics, harm reduction (digital health) service leaders and PWUD. Academics were required to have one or more peer‐reviewed articles in the harm reduction field, two or more first/corresponding author publications, and be the corresponding author of more than one article in the field of digital/digital health interventions for harm reduction, as identified from a previous scoping review. Harm reduction service leaders had to be active full‐time employees and in a management or leadership position of a digital/digital health harm reduction service for more than 6 months and have more than 5 years of experience in the harm reduction field. Individuals from the PWUD category had to identify as having current or previous experience with substance use and have previously used a supervised consumption service, in addition to having either previously responded to or experienced a drug overdose. All panellists were required to have access to an internet‐connected device to be able to complete virtual surveys. All previously identified academics and harm reduction (digital health) service leaders were contacted for recruitment; subsequently, a similar number of PWUD were contacted through relationships with previously identified academic and harm reduction service colleagues to ensure equitable representation of each participant group, with efforts to recruit international participants from diverse backgrounds. Participants were identified through purposeful sampling [26] and invited via email.

### 2.3 Procedures

#### 2.3.1 Steering committee and survey development

The initial survey was developed in conjunction with a steering committee comprised of 7 members (two from each demographic groups and one author (TM)) and the aforementioned scoping review. All members of the steering committee reviewed and approved the final survey (Table A1). All individuals on the steering committee were invited to complete the following survey rounds. Out of these members, 2/6 (both harm reduction service operators) chose to move forward into subsequent survey rounds.

#### 2.3.2 Survey

Additional participants were recruited in line with the inclusion criteria outlined above. All participants were given at least 2 weeks to provide their responses in addition to two reminder emails to ensure participant retention throughout the study. Non‐responders from previous rounds were not invited to subsequent rounds. Peer experts or people with lived/living experience of substance use were provided with $20 honoraria per round for a total of $80 for completing all 4 rounds of the survey. All participants who completed the final round of the Delphi survey were invited to review and author the final manuscript.

#### 2.3.3 Delphi Round 1

The survey consisted of open‐ended questions to ensure participants generated suggested data elements and outcome measures without external influence from the steering committee or associated scoping review (i.e., “What demographic information do you believe should be collected by these services?”) [23]. In this way, Round 1 served to generate ideas to be considered in future rounds. Qualtrics survey software was used to collect data due to the international nature of the Delphi study, the virtual nature of the services described, and to limit dominance and conformity [27].

Delphi participants were identified via the aforementioned scoping review and from word‐of‐mouth recruitment by PWUD community members. All participants completing survey rounds were invited to participate in subsequent rounds.

#### 2.3.4 Delphi Round 2

Free‐text responses collected from the previous Delphi round were summarised and transcribed by three authors (WR, DV and NS) using Excel with oversight by the principal investigator (MG) for inclusion in Round 2. All responses outside of those in which wording exactly matched those from other respondents were included in the following rounds. Additional relevant information (including terminology and outcomes measured) identified in the previously conducted scoping review [8] were also included. The various terminology and core outcome measures identified by participants from the previous round and from the scoping review were listed on a Likert scale, including “strongly disagree, disagree, neutral, agree, strongly agree and prefer not to say” in which participants were asked to rank their preferences for service terminology, potential recommended outcome variables and demographic characteristics for collection consideration. The preselected and pre‐published values of 70% agreement (i.e., selections of strongly agree or agree) or 70% disagreement (i.e., selections of strongly disagree or disagree) were required to reach a consensus consistent with previous studies [28, 29]. Consensus (>70%) was not achieved for all statements in Round 1 thus, further Delphi rounds were pursued.

**FIGURE 2:**
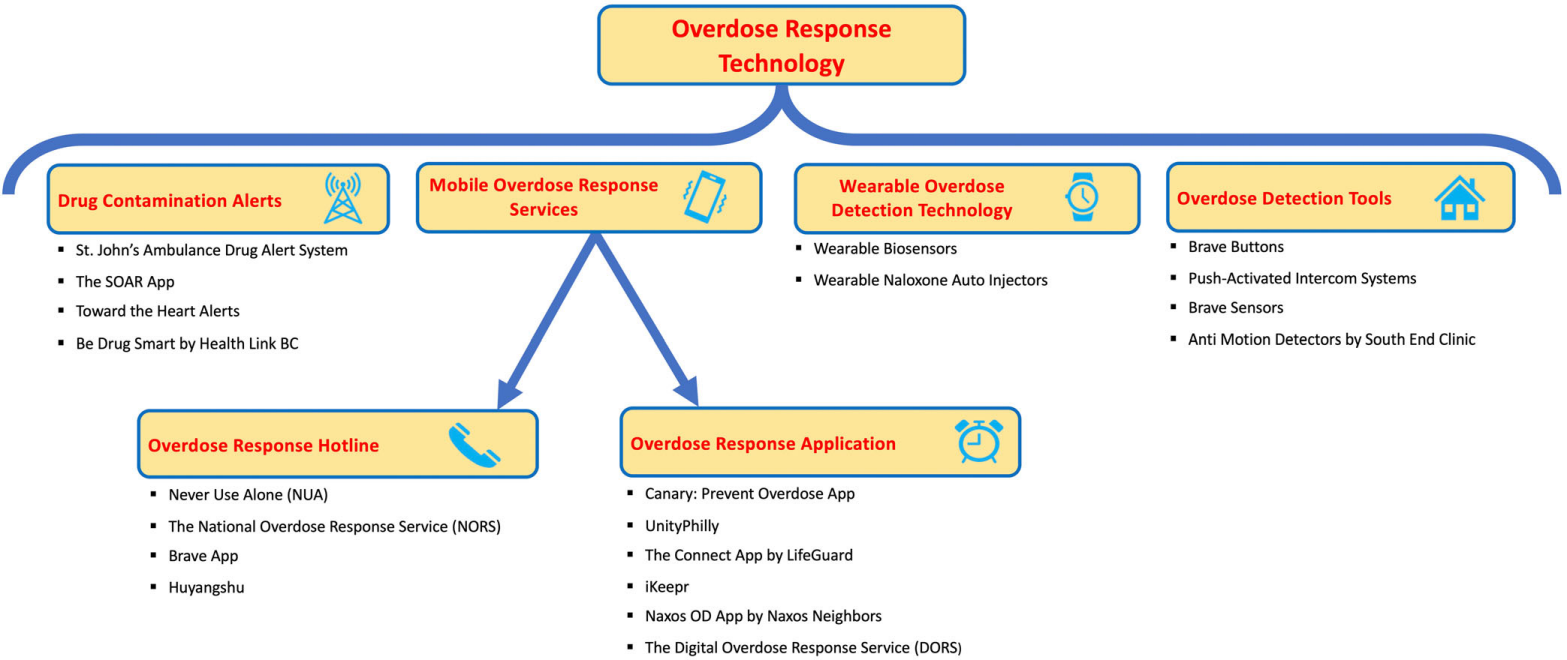
Terminology for overdose response technologies and their associated sub‐categories. *2023 overdose response technology market and may be subject to change with a rapidly updating market.

In this round, technologies and services currently available were categorised by their functionality (note attached descriptions in Figure 2). Similar to the methodology employed in Round 1, participants were asked to provide some potential names for groupings of digital health technologies and services in free form as well as any other potential services which should be included under the main umbrella terminology. Delphi consideration of recommended outcome variables and demographic characteristics that services should collect was not included in the final two rounds of the Delphi process, as items had been tallied; thus, the results for these elements of the study were considered closed after Round 2.

#### 2.3.5 Delphi Rounds 3–4

Rounds 3–4 consisted of the ranking of novel sub‐categories of terminology proposed in free form during Round 2. Round 4 of the Delphi once again required participants to rank their preferred choice of terminology for each category and subcategory to arrive at the final preferred terminology. Only terminology options that had reached consensus were included in Round 4. Each rank was assigned a value to determine a final score and the ranking of each proposed terminology that had previously reached consensus (70% agreement).

## 3 RESULTS

Of the initial 23 panellists who began the study, 14 (60.9%) completed all Delphi rounds. Geographically, 12 experts were based out of the United States, 6 from Canada and 1 participant from Germany, Italy, Spain, Israel and China, respectively. Additional participant demographics and attrition rates by participant group are summarised in Table 1.

**TABLE 1:** Demographics of panel members.

Demographics	Round 1, *N* (%)	Lost to follow‐up, *N* (%)	Completed Round 4, *N* (%)
Total	23 (100)	9 (39.1)	14 (60.9)
Age (standard deviation)	45.8 (9.1)	46.2 (8.0)	45.5 (9.6)
Gender			
Men	9 (39.1)	4 (50)	5 (35.7)
Women	12 (52.1)	4 (50)	8 (57.1)
Non‐binary	1 (4.34)	0 (0)	1 (7.14)
Ethnicity			
Caucasian	18 (78.2)	7 (77.7)	11 (78.5)
Other	5 (21.7)	2 (22.2)	3 (21.4)
Education			
Graduate degree	15 (65.2)	6 (66.6)	9 (64.2)
Bachelor's degree	4 (17.3)	0 (0)	4 (28.5)
High school degree or equivalent	3 (13.0)	3 (33.3)	0 (0)
Less than high school degree	1 (4.34)	0 (0)	1 (7.14)
Geographic region			
North America	18 (78.2)	8 (88.8)	10 (71.4)
Europe	3 (13.0)	0 (0)	3 (21.4)
Asia	2 (8.69)	1 (11.1)	1 (7.14)
Group category			
Academic	9 (39.1)	4 (44.4)	5 (35.7)
Leader/manager of service	7 (30.4)	2 (22.2)	5 (35.7)
People who use drugs	7 (30.4)	3 (33.3)	4 (28.5)
Other characteristics			
Has past experience with substance use (all groups)	10 (43.4)	4 (44.4)	6 (42.8)
Has experience responding to an overdose	17 (73.9)	6 (66.6)	11 (78.5)
Previously worked on a technology‐based harm reduction service.	13 (56.5)	4 (44.4)	9 (64.2)

### 3.1 Terminology

In the third round, three different names had reached a consensus (70% agreement), including digital harm reduction, overdose response technology and virtual overdose response services, and were considered appropriately representative of the services. The ranking was then used to identify overdose response technology as the preferred overarching terminology. Consensus from the final round is displayed in Figure 2.

### 3.2 Data collection recommendations

In terms of core outcome measurements and recommendations for data collection, 14 core demographic variables reached our a‐priori definition of consensus included: overdose events, collection of name or pseudonym as an option, neighbourhood, age, gender, past experience of overdose, ethnicity, substance used, route of administration and amount of substance used, date, time and duration of the service. Furthermore, six health outcomes were also recommended for data collection, including response type (community, emergency medical services or other), overdose events, morbidity, mortality, responder arrival times and post‐rescue care administered. Demographic items that did not reach consensus include healthcare numbers, health conditions, past mental health or substance use history, past history of violence, housing status, socioeconomic status, level of education and other lifestyle factors.

**FIGURE 3:**
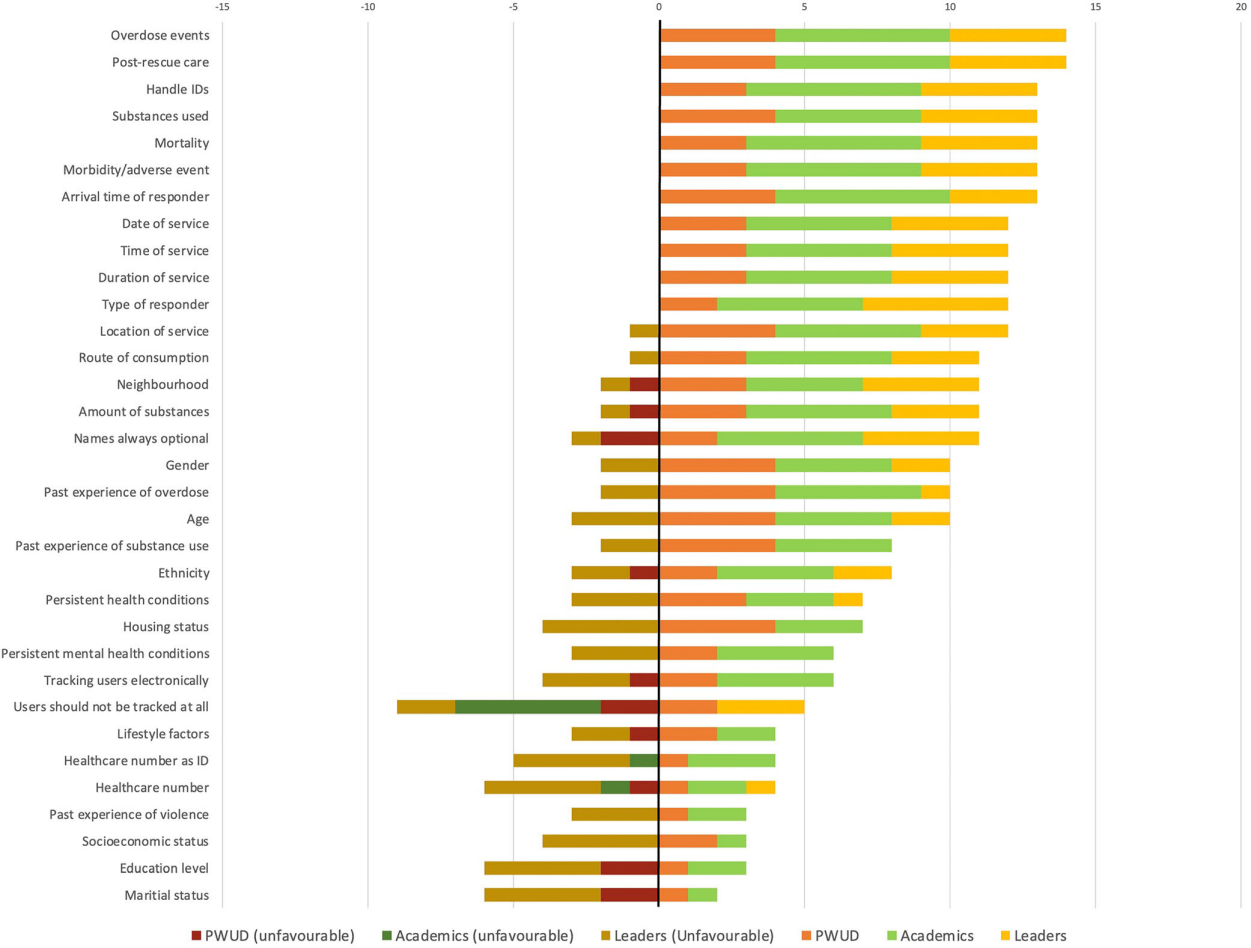
A summary of the demographic and outcomes data recommended for data collection.

Other variables initially proposed are highlighted in Figure 3. Figure 3 additionally provides a breakdown of the composition of respondents in favour versus against the collection of various demographic and outcomes data; additional data can be seen in Table A1.

## 4 DISCUSSION

This is the first international Delphi study examining both the terminology and core outcome sets associated with overdose response technologies. The terminology described herein establishes more delineated terminology for use when describing overdose response technologies. The terminology describes various technologies, such as drug contamination alerts, mobile overdose response services (comprising overdose response hotlines and overdose response applications), wearable overdose technologies and overdose detection tools. Each of these overdose response technologies is considered unique in its approach to overdose prevention, and the precise language provided will serve peers, harm reduction advocates, academics, health providers, government agencies and the general public in better communicating the categories and general capabilities of the aforementioned services. It is important to note, however, that when implementing the term mobile overdose response services throughout various manuscripts, reviewers noted overlap of this terminology with mobile supervised consumption sites, such as trailers and buses which provide safe consumption services in different areas of the same jurisdiction. After sharing this information with study participants via email, 7/10 respondents recommended the terminology be removed altogether and subheadings be used instead.

We also recommend variables that should be collected for programmatic needs and evaluation purposes. While consensus regarding collecting certain variables was achieved, data collection by harm reduction and healthcare programs may contribute to decreased uptake of services and widen access barriers for some PWUD [20]. Indeed, due to the nature of the study, our results do not include the rationales for items which reached consensus or those that did not. It can be speculated that items that did not reach consensus (such as socioeconomic status, routes of consumption, marital status, past experiences of substance use and various lifestyle factors) may be overly invasive and provide less value to groups interested in the data generated from these services. Current available services employ diverse data collection methods; current and future technologies operating in this space must therefore find a balance between data collection for evaluation and privacy for PWUD while innovating to ascertain the best ways to collect data and track service utilisation [19, 20]. Undoubtedly, due to the wide variety of overdose response technologies discussed within this manuscript, collecting some of the measures recommended by the study panellists may not be feasible. For example, overdose response buttons would likely be unable to gather specific demographic data of service users unless paired and used only by a specific user [30]. Given the importance of program evaluation to many overdose response technologies, data should be collected in a clear, consistent and defensible manner. The recommendations found within this manuscript serve as guidelines for data collection by which future studies can more accurately determine the effectiveness and uptake of services within certain populations. Aggregate data on overdose response technology service utilisation and service outcomes should be made available to the public where possible without compromising participant privacy in order for PWUDs to be able to make more informed decisions regarding their own personal safety when using substances. Data that was recommended to be collected as a minimum for the operation of the service is discussed in greater detail below:

### 4.1 Overdose events, outcomes, response times and post‐rescue care

Overdose events and response times comprised some of the minimum core outcome sets that services should aim to capture. Given that this is the most central outcome of overdose response technologies, this would enable effective assessment of overdose response technologies, but may help to better understand use patterns for PWUD. Understanding response times and related data would also enable PWUD to make informed decisions regarding their risk tolerance. For example, rural communities may see slower response times for emergency services [31, 32], and as a result, PWUD may opt for a community intervention or wearable technology to facilitate timely naloxone administration. While studies regarding these metrics continue to emerge in recent literature [30, 33], often reports, particularly those included in grey literature, lack clarity regarding their determinations of mortality prevented [33]. As a result, comments on overdose events, clearly identifying events in which naloxone was administered, would likely support comparisons of service effectiveness over time.

### 4.2 Names, pseudonyms and handle IDs

Collection of names, pseudonyms, or handle IDs was recommended for service evaluation. While there may be some direct benefits for services and service users, allowing for more personalised experiences and relationships and including these types of identifiers may also assist in additional public health monitoring in the future. For example, while yet to be studied within overdose response technologies, this may allow tracking of substance use patterns over time to determine if PWUD may modulate their service usage over time in correlation similar to data reported from supervised consumption sites [34].

### 4.3 Location‐based data collection and substances consumed

In addition to enabling assessment of the reach of overdose response technologies, collecting postal codes may allow for more timely drug contamination alerts tailored towards specific geographic areas. Furthermore, this data can inform the availability of existing harm reduction services and determine how best to support individual harm reduction needs [35, 36].

### 4.4 Gender, age and ethnicity

Gender and age‐related data were also recommended measures to be captured by overdose response technologies, while ethnicity data was not. Indeed, previous works have both hypothesised and confirmed higher usage patterns of overdose response technologies by women and gender minorities [15, 37, 38]. Where possible, age‐related data was also a recommended data collection measure, likely due to the variability in overdose rates and harm reduction service uptake across these groups [39, 40]. Previous studies of mobile health uptake by age highlight widening inequities fuelled by the inability of older individuals to adopt or use mobile health for sensitive health conditions [41]. As a result, while overdose response technologies may help decrease inequities in harm reduction service access for some populations [15, 37, 38], continued efforts should be made to target those who may not demonstrate similar levels of uptake. While not recommended by our panel for data collection, racial and ethnic minorities have shown disparities in access to harm reduction services [42] and, therefore, may present an additional population that could benefit from overdose response technologies.

### 4.5 Substance used, amount and route of administration

Additional measures recommended for collection by service providers where possible include substances used, amounts and routes of administration. Many studies have shown correlations between these various metrics and overdose risk among PWUD [43], with one overdose response technology demonstrating a similar risk of overdose and time to overdose using these metrics [37]. Furthermore, collecting these metrics would allow for the monitoring of shifting drug trends outside of brick‐and‐mortar facilities and a more rapid public health response. For example, recent trends in British Columbia, Canada, note that more than half of PWUD smoke their substances [44]; however, many of the current harm reduction supports in this region do not provide safer smoking facilities, leaving a large gap in services for this population [45].

### 4.6 Overdose events, outcomes, response times and post‐rescue care

Overdose events and response times comprised some of the minimum core outcome sets that services should aim to capture. Not only would this allow effective assessment of overdose response technologies, but it may also help to better understand use patterns for PWUD. Understanding response times and related data would also enable PWUD to make informed decisions regarding their risk tolerance. For example, rural communities may see slower response times for emergency services [31, 32] and, as a result, PWUD may opt for a community intervention or wearable technology to facilitate timely naloxone administration. While studies regarding these metrics continue to emerge in recent literature [30, 33], often reports, particularly those included in grey literature, lack clarity regarding their determinations of mortality prevented [33]. As a result, comments on overdose events, clearly identifying events in which naloxone was administered, would likely support comparisons of service effectiveness over time.

### 4.7 Strengths and limitations

One of the main methodological limitations is the number of subject experts who participated in the study and the relatively high attrition rate. The field of overdose response technology research is quite small, and, as a result, a limited number of participants were eligible to participate in the study, which may limit the generalisability of the findings. This attrition may have been compounded by significantly lower compensation for these experts, and thus future studies should consider providing additional incentives for participation in subsequent rounds. Furthermore, participants who were initially a part of the steering committee were also invited to participate in subsequent survey rounds, which may result in an over‐representation of specific ideas and perspectives among these group members. Participants were only recruited from countries which currently utilise overdose response technology and thus have more progressive drug policy and are supportive of harm reduction strategies. Participants who use drugs were most likely to be non‐responsive in subsequent rounds; however, those who completed the final version of the survey represented similar proportions of participant responses. Furthermore, while the final round of participants remained balanced between groups, attrition bias may have impacted the validity of the results. Similarly, many of the participants came from highly educated academic backgrounds, which may bias the results, especially around key metrics regarding evaluation. Lastly, due to the nature of our study, we cannot interpret the rationales for neither the items that reached consensus nor those that did not.

## 5 CONCLUSION

The results of this study outline the nomenclature recommended for the field of overdose response technologies and associate subcategories: drug contamination alerts, mobile overdose response services, overdose response hotlines and apps, wearable overdose technologies and overdose detection tools. Overdose response technologies must strike a balance between collecting data for program evaluation and the privacy and safety of PWUD. This study provides clarity on the measures that services should consider capturing for program evaluation – specifically, name or pseudonym, neighbourhood, age, gender, past experience of overdose, response type (community, emergency medical services or other), handle IDs, substance used, route of administration, amount of substance used, overdose events, morbidity, mortality, responder arrival times, post‐rescue care administered, date, time and duration of the service were all recommended. It is important to note that these findings provide recommendations and are not definitive; these findings, however, can be used to provide comparative program evaluations and guide future innovations in this field.

## AUTHOR CONTRIBUTIONS

TM and MG conceptualised the study and obtained funding for the evaluation. WR and DV collected data and wrote the manuscript. All other authors participated in manuscript review, revision and the Delphi process.

## FUNDING INFORMATION

This study was funded by a contribution from Health Canada's Substance Use and Addictions Program (SUAP Grant ID: 2122‐HQ‐000021). This study was made possible by funding from the Canadian Institute of Health Research (CIHR FRN:181006) and Grenfell Ministries. Health Canada and Grenfell Ministries had no role in the design of this study and did not have any role during its execution, analyses, interpretation of the data or decision to submit results. The views expressed herein do not necessarily represent the views of Health Canada.

## CONFLICT OF INTEREST STATEMENT

M. G. is a co‐founder and advisor for the National Overdose Response Service (NORS), receives funding from Health Canada's Substance Use and Addiction Program and is a board member of the Canadian Society of Addiction Medicine. D. V. receives salary funding through the University of Calgary via Health Canada's Substance Use and Addiction Program, which provides operational funding for NORS. S. C. receives funding from the National Institutes of Health/National Institute on Drug Abuse, National Institute on Biomedical Imaging and Bioengineering and Indivior, PLC. O. K. is a co‐founder of Brave Technology Co‐op and a co‐founder of NORS. Brave Tech Co‐op has received funding from HC SUAP for technical assistance in implementing NORS. J. C. is the Chief Executive Officer of Naxos Neighbours. M. B. is a co‐founder of Never Use Alone. All other authors declare no conflicts of interest.

## Data Availability

The data that support the findings of this study are available on request from the corresponding author. The data are not publicly available due to privacy or ethical restrictions.

## APPENDIX A

**TABLE A1:** Composition of respondents in favour versus against the collection of various demographics and outcomes.

Category	Voting item	Mean	Total respondents	Total approval	% PWUD approval	% Academic approval	% Harm reduction leader approval	Final decision
Data collection								
Methods of tracking	Users should not be tracked at all	3.0	15	5 (60%)	50	0	60	Neutral
	Users should be given the option to reveal their names or remain anonymous	3.7	15	11 (73%)	50	83	80	Recommended
	Asking users to create their own custom code that will be used to track them	4.0	14	13 (93%)	100	100	80	Recommended
	Tracking users electronically (i.e., unique phone number or email)	3.3	14	6 (43%)	50	80	0	Neutral
	Using unique health identifiers like health care numbers	2.8	14	4 (29%)	25	60	0	Neutral
Demographics	Age	3.7	13	10 (77%)	100	100	40	Recommended
	Gender	3.7	13	10 (77%)	100	100	40	Recommended
	Ethnicity	3.4	12	8 (67%)	67	100	40	Neutral
	Neighbourhood (i.e., postal/zip code)	4.0	13	11 (85%)	75	100	80	Recommended
	Education level	2.6	13	3 (23%)	25	50	0	Neutral
	Marital status	2.4	12	2 (17%)	25	33	0	Neutral
	Socioeconomic status	2.8	11	3 (27%)	67	33	0	Neutral
	Housing status (i.e., homelessness)	3.3	13	7 (54%)	100	75	0	Neutral
Health data	Healthcare number	2.6	14	4 (29%)	25	40	20	Neutral
	Lifestyle factors (i.e., smoking)	3.1	14	4 (29%)	50	40	0	Neutral
	Persistent health conditions (i.e., allergies, heart problems)	3.5	13	7 (54%)	100	60	20	Neutral
	Persistent mental health conditions (i.e. PTSD, depression)	3.3	13	6 (46%)	67	80	0	Neutral
	Past experience of substance use	3.6	14	8 (57%)	100	80	0	Neutral
	Past experience of overdose	3.7	14	10 (71%)	100	100	20	Recommended
	Past experience of violence	2.9	14	3 (21%)	25	40	0	Neutral
Substance and outcome data	Substances used	4.2	14	13 (93%)	100	83	100	Recommended
	Amount of substances	3.9	14	11 (79%)	75	83	75	Recommended
	Route of consumption	3.9	14	11 (79%)	75	83	75	Recommended
	Location of service	4.0	15	12 (80%)	100	83	60	Recommended
	Date of service	4.1	15	12 (80%)	75	83	80	Recommended
	Time of service	4.1	15	12 (80%)	75	83	80	Recommended
	Duration of service	4.1	15	12 (80%)	75	83	80	Recommended
	Overdose events	4.3	15	14 (93%)	100	100	80	Recommended
	Mortality	4.1	15	13 (87%)	75	100	80	Recommended
	Morbidity/adverse event	4.1	15	13 (87%)	75	100	80	Recommended
	Type of responder (i.e., community member, medical staff)	4.1	15	12 (80%)	50	83	100	Recommended
	Arrival time of responder	4.1	15	13 (87%)	100	100	60	Recommended
	Post‐rescue care	4.1	15	14 (93%)	100	100	80	Recommended
